# *cis*-Bis(1,10-phenanthroline-κ^2^ *N*,*N*′)bis­(pyridin-4-amine-κ*N* ^1^)ruthenium(II) bis­(hexa­fluoridophosphate)^1^

**DOI:** 10.1107/S1600536812051999

**Published:** 2013-01-09

**Authors:** Mariana R. Camilo, Felipe T. Martins, Valéria R. S. Malta, Javier Ellena, Rose M. Carlos

**Affiliations:** a Universidade Federal de São Carlos, Departamento de Química, CP 676, CEP 13565-905, São Carlos/SP, Brazil; b Universidade Federal de Goias, Instituto de Química, Campus Samambaia, CP 131, CEP 74001-970, Goiania/GO, Brazil; c Universidade Federal de Alagoas, Centro de Ciências Exatas e Naturais, Departamento de Química, CEP 57072-970, Maceió/AL, Brazil; d Universidade de São Paulo, Instituto de Física de Sao Carlos, CP 369, CEP 13560-970, São Carlos/SP, Brazil

## Abstract

In the title complex, [Ru(C_12_H_8_N_2_)_2_(C_5_H_6_N_2_)_2_](PF_6_)_2_, the Ru^II^ atom is bonded to two α-diimine ligands, *viz.* 1,10-phenanthroline (phen), in a *cis* configuration, in addition with with two 4-amino­pyridine (4Apy) ligands, resulting in a distorted octa­hedral coordination geometry. N—H⋯F hydrogen-bonding inter­actions play an important role in the crystal assembly: 2_1_-screw-axis-related complex mol­ecules and PF_6_^−^ counter-ions alternate in helical chains formed along the *a* axis by means of these contacts. N—H⋯π contacts (H⋯centroid = 3.45 Å) are responsible for cross-linking between the helical chains along [001].

## Related literature

For compounds with similar properties, see Bonneson *et al.* (1983 ▶); Salassa *et al.* (2009 ▶). For the use of 4Apy, see Sinha & Shrivastava (2012 ▶). For similar structures, see: Stoyanov *et al.* (2002 ▶).
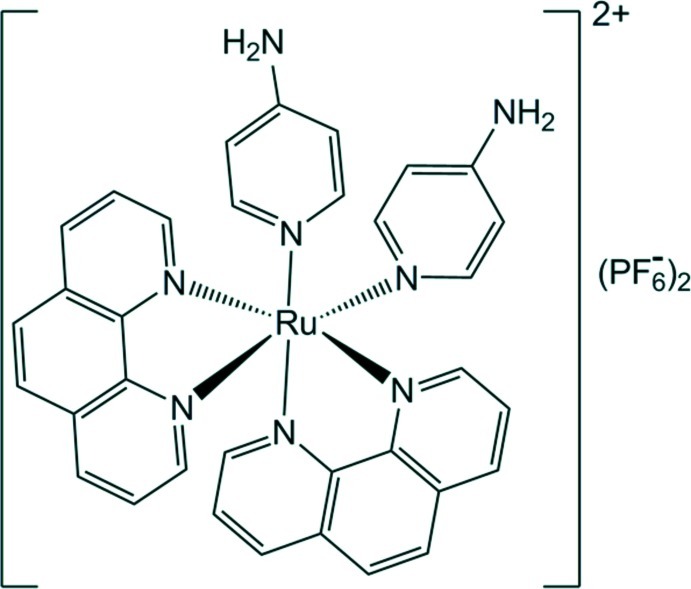


## Experimental

### 

#### Crystal data

[Ru(C_12_H_8_N_2_)_2_(C_5_H_6_N_2_)_2_](PF_6_)_2_
*M* *_r_* = 939.65
Orthorhombic, 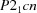

*a* = 13.0943 (3) Å
*b* = 14.5730 (3) Å
*c* = 19.9366 (5) Å
*V* = 3804.37 (15) Å^3^
*Z* = 4
Mo *K*α radiation
μ = 0.59 mm^−1^
*T* = 298 K
0.40 × 0.20 × 0.10 mm

#### Data collection

Nonius KappaCCD diffractometer
Absorption correction: Gaussian (Coppens *et al.*, 1965 ▶) *T*_min_ = 0.699, *T*_max_ = 0.938
29253 measured reflections
6964 independent reflections
5348 reflections with *I* > 2σ(*I*)
*R*_int_ = 0.066

#### Refinement

*R*[*F* ^2^ > 2σ(*F* ^2^)] = 0.053
*wR*(*F* ^2^) = 0.151
*S* = 1.04
6964 reflections
514 parameters
1 restraint
H-atom parameters constrained
Δρ_max_ = 0.39 e Å^−3^
Δρ_min_ = −0.48 e Å^−3^
Absolute structure: Flack (1983 ▶), 3264 Friedel pairs
Flack parameter: 0.25 (6)

### 

Data collection: *COLLECT* (Nonius, 2000 ▶); cell refinement: *SCALEPACK* (Otwinowski & Minor, 1997 ▶); data reduction: *DENZO* (Otwinowski & Minor, 1997 ▶) and *SCALEPACK*; program(s) used to solve structure: *SHELXS97* (Sheldrick, 2008 ▶); program(s) used to refine structure: *SHELXL97* (Sheldrick, 2008 ▶); molecular graphics: *ORTEP-3 for Windows* (Farrugia, 2012) ▶ and *Mercury* (Macrae *et al.*, 2006 ▶); software used to prepare material for publication: *WinGX* (Farrugia, 2012) ▶.

## Supplementary Material

Crystal structure: contains datablock(s) global, I. DOI: 10.1107/S1600536812051999/bg2495sup1.cif

Structure factors: contains datablock(s) I. DOI: 10.1107/S1600536812051999/bg2495Isup2.hkl

Additional supplementary materials:  crystallographic information; 3D view; checkCIF report

## Figures and Tables

**Table 1:** Hydrogen-bond geometry (Å, °)

*D*—H⋯*A*	*D*—H	H⋯*A*	*D*⋯*A*	*D*—H⋯*A*
N4—H4*A*⋯F5	0.86	2.45	3.231 (12)	151
N4*A*—H4*A*1⋯F1*A* ^i^	0.86	2.23	3.063 (12)	163
N4*A*—H4*A*2⋯F3*A* ^ii^	0.86	2.34	3.18 (2)	165

Symmetry codes: (i) 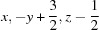; (ii) 
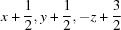
.

**Fig. 1.**
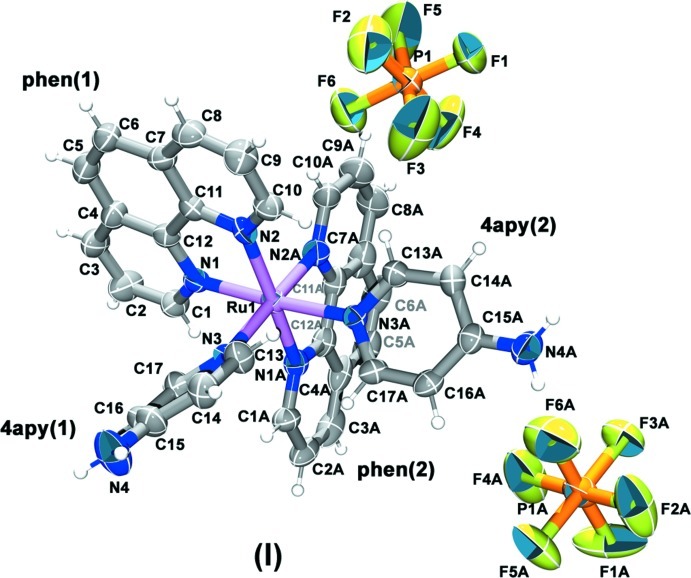
ORTEP-3 drawing of (I). Ellipsoids for non-H atoms are drawn at the 30% probability level.

**Fig. 2.**
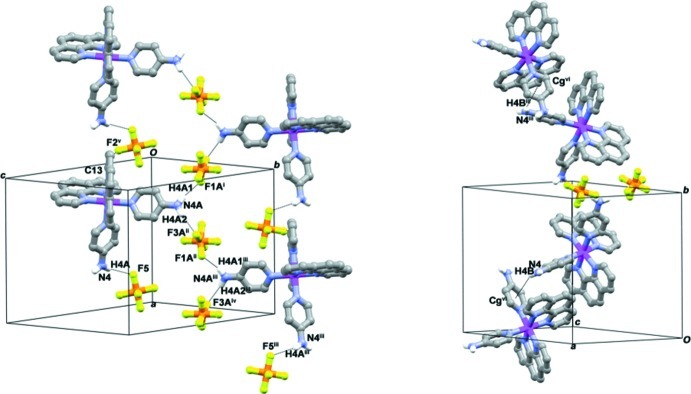
The crystal packing of (I). In this figure, a helical chain assembled along the a axis of (I) is shown on the left of the panel, while the N—H···π interaction croos-linking the helical chains paralell to the b axis is displayed on the right. Hydrogen bonds are shown as dashed lines. [Symmetry codes: (a) (i) x, -y + 3/2, z - 1/2; (ii) x + 1/2, y + 1/2, -z + 3/2; (iii) x + 1/2, -y + 2, -z + 1; (iv) x + 1, -y + 3/2, z - 1/2; (v) x - 1/2, -y + 1, -z + 1; (vi) x + 1/2, y - 1/2, -z + 3/2; (b) (i) x, y + 1, z; (ii) -x + 1, -y + 1, -z + 1; (iii) -x, -y, -z + 1; (iv) -x + 1, -y, -z; (v) -x, -y + 1, -z + 1; (vi) x - 1, y, z; (vii) x - 1, y + 1, z + 1].

## supplementary crystallographic information

### Comment

Herein we describe the crystalline structure of the complex namely, *cis*-[Ru(C_12_H_8_N_2_)_2_(C_5_H_6_N_2_)_2_](PF_6_)_2_, (I). The 4Apy complex has shown important photochemical activity toward delivery of 4Apy to the central nervous system ( Sinha & Shrivastava, 2012).

Asymmetric units of complex (I) is shown in Fig. 1. As can be seen in this figure, each Ru atom is coordinated to six nitrogen atoms from four ligand molecules. Compound (I) crystallizes in the noncentrosymmetric orthorhombic space group *P*2_1_cn, with one Ru(II) atom, two 1,10-phenanthroline (phen) ligands, two 4-aminopyridine (4Apy) and two PF_6_^-^ counterions in the asymmetric unit. Contacts of type N—H···F are also responsible for keeping coordinated ligands of (I) in contact with PF_6_ counterions (Fig. 2). In the packing of this compound, helical chains are formed along the *a* axis. 2_1_-Screw axis related complex molecules and PF_6_ counterions are alternated into these chains, which are assembled through the N4A—H4A1···F1A and N4A—H4A2···F3A contacts. A same NH_2_ group belonging to the 4Apy(2) moiety is hydrogen bonding donor in both interactions which have one of the two crystallographically independent PF_6_ units as acceptor. The other PF_6_ counterion is a hydrogen bonding acceptor from the other amine group of the 4Apy(1) moiety through the N4—H4A···F5 contact. This interaction in association with the non-classical C10A—H10A···F6 hydrogen bonding contributes to assemble the helical chains along the [100] direction. More specifically, PF_6_ units acting as hydrogen bonding acceptors through their F5 and F6 atoms are connected to translation related complex molecules of the helical backbone. At last, the other NH_2_ hydrogen of the 4Apy(1) moiety is involved in a N—H···π interaction with the π–system of the 4Apy(2) ring. The occurrence of such intermolecular contact is supported on the basis of the moderate separation between H4B and the centroid of the 4Apy(2) ring (labeled as Cg) (3.45 Å). This N4—H4B···Cg interaction is responsible for the cross-linking between helical chains along the [010] direction. Geometric parameters of the classical hydrogen bonding interactions are shown in Table 1.

The complex shows the Ru atom bonded to two a-diimine ligands in a *cis* configuration with the two 4Apy ligands in the expected distorted-octahedral fashion. The *trans* N—Ru—N angles (mean values of 176 (2)° (I), deviate slightly from the ideal value of 180°. Corresponding *cis* angles show similar small deviations from 90°: 90 (6)°. The mean Ru—*N*(α-diimine) distance [2.07 (1) Å in (I)] is similar to that found in a related coordination complex with the [Ru(bpy)_3_)]^2+^ moiety (2.056 Å) (Stoyanov *et al.*, 2002).

In the present structure, the α-diimine coordinated ligands are approximately planar with deviation from the least-square planes less than 2°. The two α-diimine ligands are nearly perpendicular, as indicated by the dihedral angle between their least square planes, 87.43 (9)° in (I).

The dihedral angles do not show any significant distortions in the structure of the complex to relieve the steric hindrance imposed by phenanthroline ligand. Fig. 1 shows a very neat twisted location of the 4Apy ligands with respect to the planes of the phen ligands. The dihedral angles between the least-squares planes calculated through 4Apy and phen ligands are 86.9 (1)° [4Apy(1) and phen(1)] and 79.2 (1)° [4Apy(2) and phen(2)].

### Experimental

The compound (I) was synthesized from the corresponding aquo-complex (Bonneson *et al.*, 1983) *cis*-[Ru(α-diimine)_2_(OH_2_)_2_](PF_6_)_2_ [α-diimine = 1,10-phenanthroline (phen)] by reacting the latter with 4-Aminopyridine in 1:1 EtOH/H_2_O mixture under nitrogen atmosphere and for 8 h under reflux. A stoichiometric amount of ammonium hexafluorophosphate was added to precipitate the complex. The resulting solid products were crystallized from acetonitrile. Elemental analysis (%) for (I) RuC_34_H_32_N_8_P_2_F_12_O_2_: calculated: C, 41.80, N, 11.48; H, 3.30; found: C, 41.7; N, 11.5; H, 3.13.8; found: C, 38.70; N, 11.80; H, 3.54.

### Refinement

The H atoms were located from the difference Fourier synthesis and refined using the riding model on their parent atoms with C—H = 0.93 Å for aromatic moieties or 0.96 Å for methyl group of acetonitrile, N—H = 0.86 Å and *U*_iso_(H) = 1.2*U*_eq_ for phenyl and amine H atoms or 1.5*U*_eq_ for methyl ones.

### Crystal data

**Table:**

[Ru(C_12_H_8_N_2_)_2_(C_5_H_6_N_2_)_2_](PF_6_)_2_	*F*(000) = 1880
*M**_r_* = 939.65	*D*_x_ = 1.641 Mg m^−^^3^
Orthorhombic, *P*2_1_*c**n*	Mo *K*α radiation, λ = 0.71073 Å
Hall symbol: P -2n 2a	Cell parameters from 35299 reflections
*a* = 13.0943 (3) Å	θ = 2.9–25.7°
*b* = 14.5730 (3) Å	µ = 0.59 mm^−^^1^
*c* = 19.9366 (5) Å	*T* = 298 K
*V* = 3804.37 (15) Å^3^	Prism, red
*Z* = 4	0.40 × 0.20 × 0.10 mm

### Data collection

**Table:**

Nonius KappaCCD diffractometer	5348 reflections with *I* > 2σ(*I*)
CCD scans	*R*_int_ = 0.066
Absorption correction: gaussian (Coppens *et al.*, 1965)	θ_max_ = 25.7°, θ_min_ = 2.9°
*T*_min_ = 0.699, *T*_max_ = 0.938	*h* = −15→15
29253 measured reflections	*k* = −17→17
6964 independent reflections	*l* = −24→24

### Refinement

**Table:**

Refinement on *F*^2^	H-atom parameters constrained
Least-squares matrix: full	*w* = 1/[σ^2^(*F*_o_^2^) + (0.0819*P*)^2^ + 2.252*P*] where *P* = (*F*_o_^2^ + 2*F*_c_^2^)/3
*R*[*F*^2^ > 2σ(*F*^2^)] = 0.053	(Δ/σ)_max_ = 0.001
*wR*(*F*^2^) = 0.151	Δρ_max_ = 0.39 e Å^−^^3^
*S* = 1.04	Δρ_min_ = −0.48 e Å^−^^3^
6964 reflections	Absolute structure: Flack (1983), 3264 Friedel pairs
514 parameters	Flack parameter: 0.25 (6)
1 restraint	

### Special details

**Table:**

Geometry. All e.s.d.'s (except the e.s.d. in the dihedral angle between two l.s. planes) are estimated using the full covariance matrix. The cell e.s.d.'s are taken into account individually in the estimation of e.s.d.'s in distances, angles and torsion angles; correlations between e.s.d.'s in cell parameters are only used when they are defined by crystal symmetry. An approximate (isotropic) treatment of cell e.s.d.'s is used for estimating e.s.d.'s involving l.s. planes.

### Fractional atomic coordinates and isotropic or equivalent isotropic displacement parameters (Å^2^)

**Table:**

	*x*	*y*	*z*	*U*_iso_*/*U*_eq_	
Ru1	0.15387 (9)	0.53833 (3)	0.74946 (2)	0.05771 (15)	
P1A	−0.07388 (19)	0.48572 (17)	1.00634 (11)	0.0984 (7)	
P1	0.80453 (18)	0.52929 (14)	0.54965 (10)	0.0872 (5)	
N3A	0.1641 (4)	0.6513 (3)	0.6842 (2)	0.0607 (11)	
N1	0.1410 (4)	0.4247 (3)	0.8099 (2)	0.0702 (12)	
N1A	0.1800 (3)	0.6329 (3)	0.8261 (2)	0.0623 (13)	
F3	0.9191 (5)	0.5450 (5)	0.5394 (5)	0.185 (4)	
N3	0.3077 (4)	0.5015 (3)	0.7311 (2)	0.0581 (10)	
C13A	0.0842 (5)	0.6831 (4)	0.6485 (4)	0.0740 (16)	
H13A	0.0243	0.6488	0.6486	0.089*	
C11A	−0.0013 (5)	0.6384 (4)	0.8232 (3)	0.0651 (14)	
C11	0.0970 (5)	0.3550 (4)	0.7056 (3)	0.0714 (15)	
C7A	−0.0949 (5)	0.6733 (5)	0.8473 (3)	0.0816 (18)	
C12A	0.0923 (5)	0.6686 (4)	0.8520 (3)	0.0659 (14)	
F6	0.8266 (5)	0.4546 (3)	0.6050 (3)	0.1222 (17)	
C6A	−0.0927 (7)	0.7386 (5)	0.8989 (4)	0.094 (2)	
H6A	−0.1547	0.762	0.9139	0.112*	
C17A	0.2501 (5)	0.7059 (4)	0.6828 (4)	0.0773 (17)	
H17A	0.3069	0.6883	0.7078	0.093*	
C4A	0.0903 (6)	0.7348 (4)	0.9049 (3)	0.0729 (16)	
C12	0.1115 (5)	0.3467 (4)	0.7771 (4)	0.0769 (17)	
F1	0.7817 (4)	0.6041 (5)	0.4944 (3)	0.150 (3)	
C5A	−0.0065 (6)	0.7699 (5)	0.9284 (4)	0.088 (2)	
H5A	−0.0095	0.8127	0.9629	0.105*	
C1	0.1573 (7)	0.4181 (5)	0.8760 (3)	0.0878 (18)	
H1	0.1749	0.4706	0.8999	0.105*	
C2A	0.2720 (6)	0.7330 (5)	0.9000 (4)	0.095 (2)	
H2A	0.3347	0.7555	0.9145	0.115*	
C14A	0.0856 (6)	0.7631 (5)	0.6119 (3)	0.0773 (17)	
H14A	0.0282	0.7805	0.5876	0.093*	
C9	0.0738 (5)	0.3750 (6)	0.5722 (4)	0.091 (2)	
H9	0.0674	0.3827	0.5261	0.109*	
N2A	0.0059 (4)	0.5751 (4)	0.7731 (2)	0.0637 (11)	
F2	0.8036 (11)	0.4537 (5)	0.4968 (3)	0.214 (5)	
C15A	0.1706 (7)	0.8169 (5)	0.6111 (3)	0.090 (2)	
C9A	−0.1744 (6)	0.5747 (8)	0.7679 (5)	0.107 (3)	
H9A	−0.2332	0.552	0.7477	0.128*	
F5	0.6885 (5)	0.5172 (7)	0.5623 (5)	0.210 (4)	
N2	0.1136 (4)	0.4397 (4)	0.6788 (3)	0.0670 (12)	
F4	0.8079 (10)	0.6051 (5)	0.6041 (4)	0.216 (4)	
C1A	0.2674 (6)	0.6659 (5)	0.8500 (3)	0.0759 (18)	
H1A	0.3283	0.6433	0.8325	0.091*	
C3A	0.1856 (6)	0.7641 (5)	0.9268 (4)	0.087 (2)	
H3A	0.1892	0.8069	0.9613	0.105*	
N4A	0.1747 (7)	0.8976 (5)	0.5778 (4)	0.140 (3)	
H4A1	0.1223	0.9169	0.556	0.167*	
H4A2	0.2298	0.9298	0.5785	0.167*	
C8A	−0.1830 (6)	0.6385 (6)	0.8176 (4)	0.096 (2)	
H8A	−0.247	0.6585	0.8316	0.115*	
C16A	0.2548 (6)	0.7859 (5)	0.6457 (4)	0.088 (2)	
H16A	0.3154	0.819	0.6441	0.106*	
C4	0.0975 (6)	0.2625 (5)	0.8085 (5)	0.101 (3)	
C3	0.1177 (8)	0.2590 (7)	0.8792 (5)	0.117 (3)	
H3	0.1094	0.2046	0.903	0.14*	
C10A	−0.0813 (6)	0.5436 (5)	0.7473 (3)	0.080 (2)	
H10A	−0.0786	0.4989	0.7141	0.096*	
C6	0.0501 (6)	0.1941 (6)	0.7015 (6)	0.101 (3)	
H6	0.0289	0.1434	0.6769	0.121*	
C5	0.0644 (7)	0.1858 (6)	0.7664 (7)	0.118 (3)	
H5	0.0531	0.129	0.7864	0.141*	
C10	0.1018 (5)	0.4487 (5)	0.6122 (3)	0.0739 (16)	
H10	0.1126	0.5057	0.5925	0.089*	
C2	0.1486 (10)	0.3352 (7)	0.9097 (4)	0.119 (3)	
H2	0.1649	0.3329	0.9551	0.143*	
C13	0.3510 (5)	0.5010 (4)	0.6701 (3)	0.0665 (14)	
H13	0.3114	0.5193	0.6338	0.08*	
C17	0.3694 (5)	0.4711 (4)	0.7810 (4)	0.0706 (15)	
H17	0.3423	0.4695	0.8241	0.085*	
C15	0.5118 (5)	0.4449 (4)	0.7086 (3)	0.0720 (16)	
C16	0.4683 (5)	0.4424 (5)	0.7732 (4)	0.0763 (17)	
H16	0.506	0.4218	0.8098	0.092*	
C14	0.4488 (5)	0.4756 (4)	0.6583 (3)	0.0748 (16)	
H14	0.474	0.479	0.6148	0.09*	
N4	0.6095 (5)	0.4218 (5)	0.6990 (3)	0.1020 (19)	
H4A	0.6361	0.4258	0.6597	0.122*	
H4B	0.6458	0.4029	0.7322	0.122*	
C8	0.0556 (6)	0.2912 (7)	0.6005 (6)	0.112 (3)	
H8	0.0357	0.2419	0.5739	0.134*	
C7	0.0666 (5)	0.2803 (5)	0.6678 (5)	0.091 (2)	
F1A	0.0196 (7)	0.5302 (7)	0.9756 (4)	0.208 (4)	
F6A	−0.1797 (7)	0.4604 (5)	1.0366 (5)	0.171 (3)	
F2A	−0.0723 (8)	0.5749 (5)	1.0464 (5)	0.220 (4)	
F3A	−0.1282 (16)	0.5199 (11)	0.9488 (7)	0.419 (15)	
F5A	−0.0223 (11)	0.4465 (7)	1.0632 (7)	0.289 (8)	
F4A	−0.0741 (7)	0.3944 (6)	0.9729 (6)	0.237 (5)	

### Atomic displacement parameters (Å^2^)

**Table:**

	*U*^11^	*U*^22^	*U*^33^	*U*^12^	*U*^13^	*U*^23^
Ru1	0.0572 (2)	0.0581 (2)	0.0578 (2)	−0.0022 (3)	−0.00123 (18)	0.00524 (17)
P1A	0.1151 (19)	0.1057 (16)	0.0743 (11)	−0.0329 (15)	0.0118 (12)	0.0029 (11)
P1	0.0829 (12)	0.1037 (13)	0.0751 (11)	−0.0042 (11)	−0.0063 (10)	0.0133 (10)
N3A	0.061 (3)	0.068 (2)	0.054 (2)	−0.003 (3)	−0.006 (2)	0.0084 (18)
N1	0.068 (3)	0.072 (3)	0.071 (3)	−0.003 (3)	0.012 (3)	0.011 (2)
N1A	0.056 (3)	0.060 (3)	0.070 (3)	−0.001 (2)	−0.013 (2)	0.007 (2)
F3	0.082 (4)	0.187 (6)	0.287 (10)	−0.019 (4)	−0.017 (5)	0.113 (6)
N3	0.062 (3)	0.056 (3)	0.056 (2)	0.003 (2)	−0.002 (2)	0.002 (2)
C13A	0.067 (4)	0.070 (4)	0.085 (4)	−0.001 (3)	−0.012 (3)	0.006 (3)
C11A	0.071 (4)	0.068 (3)	0.056 (3)	−0.003 (3)	0.003 (3)	0.007 (3)
C11	0.056 (3)	0.063 (3)	0.096 (4)	−0.003 (3)	0.015 (3)	−0.003 (3)
C7A	0.077 (4)	0.088 (4)	0.080 (4)	0.013 (3)	0.013 (3)	0.001 (3)
C12A	0.067 (4)	0.069 (3)	0.062 (3)	0.001 (3)	−0.009 (3)	0.009 (3)
F6	0.146 (5)	0.115 (4)	0.106 (3)	−0.005 (3)	−0.021 (3)	0.023 (3)
C6A	0.098 (6)	0.085 (5)	0.098 (5)	0.019 (4)	0.023 (5)	0.000 (4)
C17A	0.067 (4)	0.073 (4)	0.092 (5)	−0.008 (3)	0.006 (3)	0.023 (3)
C4A	0.094 (5)	0.066 (3)	0.059 (3)	0.001 (3)	−0.001 (3)	−0.004 (3)
C12	0.063 (3)	0.062 (3)	0.106 (5)	0.002 (3)	0.026 (4)	0.010 (4)
F1	0.109 (4)	0.184 (5)	0.158 (5)	−0.041 (4)	−0.049 (3)	0.096 (4)
C5A	0.103 (6)	0.081 (4)	0.080 (4)	0.015 (4)	0.007 (4)	−0.002 (3)
C1	0.098 (5)	0.094 (4)	0.072 (4)	0.022 (5)	0.024 (4)	0.022 (3)
C2A	0.081 (5)	0.095 (5)	0.111 (6)	−0.012 (4)	−0.007 (4)	−0.010 (4)
C14A	0.082 (4)	0.090 (4)	0.061 (3)	0.017 (4)	0.001 (3)	0.014 (3)
C9	0.066 (4)	0.116 (6)	0.090 (5)	−0.002 (4)	−0.004 (3)	−0.043 (4)
N2A	0.060 (3)	0.075 (3)	0.057 (2)	−0.002 (2)	−0.003 (2)	0.003 (2)
F2	0.405 (16)	0.149 (6)	0.087 (4)	−0.094 (7)	0.001 (6)	−0.025 (4)
C15A	0.103 (6)	0.085 (4)	0.082 (4)	0.011 (4)	0.008 (4)	0.030 (3)
C9A	0.062 (4)	0.155 (8)	0.103 (5)	−0.017 (5)	0.001 (4)	−0.027 (6)
F5	0.095 (4)	0.291 (10)	0.243 (8)	0.007 (5)	0.022 (5)	0.141 (8)
N2	0.054 (3)	0.071 (3)	0.076 (3)	−0.001 (2)	0.004 (2)	0.002 (2)
F4	0.362 (14)	0.132 (5)	0.155 (6)	0.047 (7)	−0.047 (7)	−0.028 (5)
C1A	0.079 (5)	0.076 (4)	0.073 (4)	−0.003 (3)	−0.013 (3)	−0.004 (3)
C3A	0.114 (7)	0.074 (4)	0.074 (4)	−0.008 (4)	−0.013 (4)	−0.014 (3)
N4A	0.125 (7)	0.123 (6)	0.170 (7)	0.009 (5)	0.004 (5)	0.077 (5)
C8A	0.070 (4)	0.129 (6)	0.089 (5)	0.017 (4)	0.008 (4)	−0.012 (5)
C16A	0.085 (5)	0.072 (4)	0.109 (5)	0.000 (4)	0.012 (4)	0.017 (4)
C4	0.086 (5)	0.066 (4)	0.150 (8)	−0.007 (4)	0.041 (5)	0.015 (4)
C3	0.134 (8)	0.102 (6)	0.114 (6)	0.008 (5)	0.033 (5)	0.056 (5)
C10A	0.060 (4)	0.099 (6)	0.080 (5)	−0.005 (3)	−0.006 (3)	−0.015 (3)
C6	0.084 (5)	0.074 (5)	0.144 (8)	−0.011 (4)	0.029 (5)	−0.015 (5)
C5	0.106 (7)	0.059 (4)	0.187 (10)	−0.017 (4)	0.056 (7)	0.001 (6)
C10	0.074 (4)	0.084 (4)	0.063 (3)	−0.002 (3)	0.001 (3)	−0.010 (3)
C2	0.151 (8)	0.112 (6)	0.095 (5)	0.009 (7)	0.032 (6)	0.044 (5)
C13	0.068 (4)	0.073 (4)	0.059 (3)	−0.009 (3)	0.005 (3)	−0.006 (3)
C17	0.070 (4)	0.078 (4)	0.064 (4)	0.005 (3)	0.000 (3)	0.006 (3)
C15	0.067 (4)	0.066 (3)	0.083 (4)	0.001 (3)	0.002 (3)	−0.009 (3)
C16	0.065 (4)	0.072 (4)	0.093 (4)	0.010 (3)	−0.002 (3)	0.002 (3)
C14	0.081 (4)	0.075 (4)	0.068 (4)	−0.005 (3)	0.006 (3)	−0.019 (3)
N4	0.078 (4)	0.130 (5)	0.098 (4)	0.013 (4)	0.008 (3)	−0.024 (4)
C8	0.065 (4)	0.106 (6)	0.164 (9)	−0.008 (4)	−0.008 (5)	−0.075 (7)
C7	0.050 (4)	0.076 (4)	0.147 (8)	−0.005 (3)	0.006 (4)	−0.031 (5)
F1A	0.162 (7)	0.296 (11)	0.167 (7)	−0.117 (7)	0.074 (6)	−0.047 (6)
F6A	0.150 (6)	0.179 (7)	0.184 (7)	−0.042 (4)	0.052 (6)	0.002 (5)
F2A	0.199 (8)	0.173 (6)	0.289 (10)	−0.035 (6)	−0.024 (8)	−0.118 (7)
F3A	0.54 (3)	0.429 (19)	0.291 (13)	−0.312 (19)	−0.279 (17)	0.282 (14)
F5A	0.361 (17)	0.187 (7)	0.320 (14)	−0.075 (8)	−0.219 (13)	0.112 (8)
F4A	0.174 (7)	0.205 (7)	0.333 (12)	−0.027 (6)	0.088 (8)	−0.153 (8)

### Geometric parameters (Å, º)

**Table:**

Ru1—N1	2.054 (5)	C1—H1	0.93
Ru1—N2A	2.065 (5)	C2A—C3A	1.330 (10)
Ru1—N2	2.081 (5)	C2A—C1A	1.399 (10)
Ru1—N1A	2.086 (5)	C2A—H2A	0.93
Ru1—N3A	2.103 (4)	C14A—C15A	1.361 (11)
Ru1—N3	2.117 (5)	C14A—H14A	0.93
P1A—F5A	1.438 (9)	C9—C8	1.367 (13)
P1A—F3A	1.439 (9)	C9—C10	1.386 (9)
P1A—F4A	1.488 (7)	C9—H9	0.93
P1A—F1A	1.515 (7)	N2A—C10A	1.334 (9)
P1A—F2A	1.526 (7)	C15A—N4A	1.353 (9)
P1A—F6A	1.556 (8)	C15A—C16A	1.377 (11)
P1—F2	1.525 (6)	C9A—C8A	1.363 (12)
P1—F3	1.531 (7)	C9A—C10A	1.364 (12)
P1—F4	1.549 (7)	C9A—H9A	0.93
P1—F5	1.550 (7)	N2—C10	1.342 (8)
P1—F6	1.577 (5)	C1A—H1A	0.93
P1—F1	1.579 (5)	C3A—H3A	0.93
N3A—C13A	1.347 (8)	N4A—H4A1	0.86
N3A—C17A	1.379 (8)	N4A—H4A2	0.86
N1—C1	1.338 (8)	C8A—H8A	0.93
N1—C12	1.367 (8)	C16A—H16A	0.93
N1A—C1A	1.329 (8)	C4—C3	1.435 (14)
N1A—C12A	1.363 (8)	C4—C5	1.464 (13)
N3—C13	1.341 (7)	C3—C2	1.331 (13)
N3—C17	1.355 (8)	C3—H3	0.93
C13A—C14A	1.375 (9)	C10A—H10A	0.93
C13A—H13A	0.93	C6—C5	1.312 (14)
C11A—N2A	1.364 (8)	C6—C7	1.442 (12)
C11A—C7A	1.411 (9)	C6—H6	0.93
C11A—C12A	1.423 (9)	C5—H5	0.93
C11—N2	1.363 (8)	C10—H10	0.93
C11—C7	1.383 (10)	C2—H2	0.93
C11—C12	1.442 (11)	C13—C14	1.353 (9)
C7A—C8A	1.392 (10)	C13—H13	0.93
C7A—C6A	1.402 (10)	C17—C16	1.370 (9)
C12A—C4A	1.430 (9)	C17—H17	0.93
C6A—C5A	1.351 (11)	C15—N4	1.336 (10)
C6A—H6A	0.93	C15—C14	1.373 (10)
C17A—C16A	1.382 (9)	C15—C16	1.410 (10)
C17A—H17A	0.93	C16—H16	0.93
C4A—C3A	1.389 (11)	C14—H14	0.93
C4A—C5A	1.446 (10)	N4—H4A	0.86
C12—C4	1.389 (9)	N4—H4B	0.86
C5A—H5A	0.93	C8—C7	1.358 (13)
C1—C2	1.388 (10)	C8—H8	0.93
			
N1—Ru1—N2A	89.9 (2)	C4A—C5A—H5A	120.9
N1—Ru1—N2	79.6 (2)	N1—C1—C2	121.8 (8)
N2A—Ru1—N2	95.5 (2)	N1—C1—H1	119.1
N1—Ru1—N1A	96.70 (19)	C2—C1—H1	119.1
N2A—Ru1—N1A	79.39 (19)	C3A—C2A—C1A	119.2 (7)
N2—Ru1—N1A	173.76 (19)	C3A—C2A—H2A	120.4
N1—Ru1—N3A	177.5 (2)	C1A—C2A—H2A	120.4
N2A—Ru1—N3A	89.9 (2)	C15A—C14A—C13A	120.3 (6)
N2—Ru1—N3A	97.90 (18)	C15A—C14A—H14A	119.8
N1A—Ru1—N3A	85.74 (18)	C13A—C14A—H14A	119.8
N1—Ru1—N3	88.6 (2)	C8—C9—C10	120.1 (8)
N2A—Ru1—N3	176.74 (18)	C8—C9—H9	120
N2—Ru1—N3	87.06 (19)	C10—C9—H9	120
N1A—Ru1—N3	97.92 (18)	C10A—N2A—C11A	117.1 (6)
N3A—Ru1—N3	91.8 (2)	C10A—N2A—Ru1	128.8 (4)
F5A—P1A—F3A	176.7 (7)	C11A—N2A—Ru1	114.0 (4)
F5A—P1A—F4A	90.0 (7)	N4A—C15A—C14A	122.7 (8)
F3A—P1A—F4A	87.2 (9)	N4A—C15A—C16A	119.9 (8)
F5A—P1A—F1A	96.3 (8)	C14A—C15A—C16A	117.4 (6)
F3A—P1A—F1A	85.9 (8)	C8A—C9A—C10A	121.3 (7)
F4A—P1A—F1A	101.7 (5)	C8A—C9A—H9A	119.4
F5A—P1A—F2A	85.4 (7)	C10A—C9A—H9A	119.4
F3A—P1A—F2A	97.4 (9)	C10—N2—C11	117.3 (6)
F4A—P1A—F2A	175.1 (7)	C10—N2—Ru1	129.2 (4)
F1A—P1A—F2A	80.6 (5)	C11—N2—Ru1	113.6 (4)
F5A—P1A—F6A	91.1 (8)	N1A—C1A—C2A	123.0 (7)
F3A—P1A—F6A	87.2 (9)	N1A—C1A—H1A	118.5
F4A—P1A—F6A	87.7 (4)	C2A—C1A—H1A	118.5
F1A—P1A—F6A	168.0 (6)	C2A—C3A—C4A	122.2 (7)
F2A—P1A—F6A	90.6 (5)	C2A—C3A—H3A	118.9
F2—P1—F3	91.3 (6)	C4A—C3A—H3A	118.9
F2—P1—F4	178.6 (7)	C15A—N4A—H4A1	120
F3—P1—F4	87.7 (6)	C15A—N4A—H4A2	120
F2—P1—F5	91.3 (7)	H4A1—N4A—H4A2	120
F3—P1—F5	177.4 (6)	C9A—C8A—C7A	119.3 (7)
F4—P1—F5	89.7 (7)	C9A—C8A—H8A	120.4
F2—P1—F6	89.2 (4)	C7A—C8A—H8A	120.4
F3—P1—F6	91.0 (3)	C15A—C16A—C17A	120.6 (7)
F4—P1—F6	89.8 (4)	C15A—C16A—H16A	119.7
F5—P1—F6	89.3 (4)	C17A—C16A—H16A	119.7
F2—P1—F1	90.8 (4)	C12—C4—C3	116.7 (8)
F3—P1—F1	89.4 (3)	C12—C4—C5	117.1 (9)
F4—P1—F1	90.1 (4)	C3—C4—C5	126.1 (8)
F5—P1—F1	90.3 (3)	C2—C3—C4	118.4 (7)
F6—P1—F1	179.6 (4)	C2—C3—H3	120.8
C13A—N3A—C17A	115.2 (5)	C4—C3—H3	120.8
C13A—N3A—Ru1	123.2 (4)	N2A—C10A—C9A	122.3 (7)
C17A—N3A—Ru1	121.0 (4)	N2A—C10A—H10A	118.8
C1—N1—C12	117.1 (6)	C9A—C10A—H10A	118.8
C1—N1—Ru1	128.5 (5)	C5—C6—C7	121.3 (8)
C12—N1—Ru1	114.4 (4)	C5—C6—H6	119.4
C1A—N1A—C12A	116.9 (5)	C7—C6—H6	119.4
C1A—N1A—Ru1	130.0 (4)	C6—C5—C4	122.5 (8)
C12A—N1A—Ru1	113.0 (4)	C6—C5—H5	118.8
C13—N3—C17	114.2 (6)	C4—C5—H5	118.8
C13—N3—Ru1	124.1 (4)	N2—C10—C9	121.6 (7)
C17—N3—Ru1	121.6 (4)	N2—C10—H10	119.2
N3A—C13A—C14A	124.2 (6)	C9—C10—H10	119.2
N3A—C13A—H13A	117.9	C3—C2—C1	122.0 (8)
C14A—C13A—H13A	117.9	C3—C2—H2	119
N2A—C11A—C7A	123.6 (6)	C1—C2—H2	119
N2A—C11A—C12A	116.5 (6)	N3—C13—C14	124.0 (6)
C7A—C11A—C12A	119.9 (6)	N3—C13—H13	118
N2—C11—C7	123.0 (7)	C14—C13—H13	118
N2—C11—C12	116.3 (5)	N3—C17—C16	125.5 (7)
C7—C11—C12	120.7 (6)	N3—C17—H17	117.2
C8A—C7A—C6A	125.2 (7)	C16—C17—H17	117.2
C8A—C7A—C11A	116.3 (6)	N4—C15—C14	123.6 (7)
C6A—C7A—C11A	118.5 (7)	N4—C15—C16	120.7 (7)
N1A—C12A—C11A	117.1 (5)	C14—C15—C16	115.6 (6)
N1A—C12A—C4A	123.5 (6)	C17—C16—C15	118.5 (7)
C11A—C12A—C4A	119.4 (6)	C17—C16—H16	120.7
C5A—C6A—C7A	124.5 (7)	C15—C16—H16	120.7
C5A—C6A—H6A	117.8	C13—C14—C15	122.0 (6)
C7A—C6A—H6A	117.8	C13—C14—H14	119
N3A—C17A—C16A	122.2 (6)	C15—C14—H14	119
N3A—C17A—H17A	118.9	C15—N4—H4A	120
C16A—C17A—H17A	118.9	C15—N4—H4B	120
C3A—C4A—C12A	115.0 (7)	H4A—N4—H4B	120
C3A—C4A—C5A	125.3 (6)	C7—C8—C9	119.5 (7)
C12A—C4A—C5A	119.6 (7)	C7—C8—H8	120.2
N1—C12—C4	123.8 (8)	C9—C8—H8	120.2
N1—C12—C11	116.1 (5)	C8—C7—C11	118.5 (8)
C4—C12—C11	120.1 (7)	C8—C7—C6	123.1 (8)
C6A—C5A—C4A	118.1 (7)	C11—C7—C6	118.3 (9)
C6A—C5A—H5A	120.9		
			
N2A—Ru1—N3A—C13A	−33.7 (5)	C12A—C11A—N2A—Ru1	−0.7 (6)
N2—Ru1—N3A—C13A	61.8 (5)	N1—Ru1—N2A—C10A	−81.1 (6)
N1A—Ru1—N3A—C13A	−113.1 (5)	N2—Ru1—N2A—C10A	−1.6 (6)
N3—Ru1—N3A—C13A	149.1 (5)	N1A—Ru1—N2A—C10A	−178.0 (6)
N2A—Ru1—N3A—C17A	136.6 (5)	N3A—Ru1—N2A—C10A	96.4 (6)
N2—Ru1—N3A—C17A	−127.9 (5)	N1—Ru1—N2A—C11A	97.0 (4)
N1A—Ru1—N3A—C17A	57.2 (5)	N2—Ru1—N2A—C11A	176.5 (4)
N3—Ru1—N3A—C17A	−40.6 (5)	N1A—Ru1—N2A—C11A	0.1 (4)
N2A—Ru1—N1—C1	−85.9 (7)	N3A—Ru1—N2A—C11A	−85.6 (4)
N2—Ru1—N1—C1	178.5 (7)	C13A—C14A—C15A—N4A	−177.8 (8)
N1A—Ru1—N1—C1	−6.6 (7)	C13A—C14A—C15A—C16A	2.7 (11)
N3—Ru1—N1—C1	91.2 (7)	C7—C11—N2—C10	−1.6 (9)
N2A—Ru1—N1—C12	93.8 (5)	C12—C11—N2—C10	179.2 (6)
N2—Ru1—N1—C12	−1.8 (4)	C7—C11—N2—Ru1	178.0 (5)
N1A—Ru1—N1—C12	173.1 (4)	C12—C11—N2—Ru1	−1.1 (6)
N3—Ru1—N1—C12	−89.1 (5)	N1—Ru1—N2—C10	−178.8 (6)
N1—Ru1—N1A—C1A	95.8 (6)	N2A—Ru1—N2—C10	92.3 (6)
N2A—Ru1—N1A—C1A	−175.5 (6)	N3A—Ru1—N2—C10	1.6 (6)
N3A—Ru1—N1A—C1A	−84.9 (5)	N3—Ru1—N2—C10	−89.7 (6)
N3—Ru1—N1A—C1A	6.3 (6)	N1—Ru1—N2—C11	1.6 (4)
N1—Ru1—N1A—C12A	−88.2 (4)	N2A—Ru1—N2—C11	−87.3 (4)
N2A—Ru1—N1A—C12A	0.5 (4)	N3A—Ru1—N2—C11	−178.0 (4)
N3A—Ru1—N1A—C12A	91.2 (4)	N3—Ru1—N2—C11	90.7 (4)
N3—Ru1—N1A—C12A	−177.6 (4)	C12A—N1A—C1A—C2A	1.0 (9)
N1—Ru1—N3—C13	134.1 (5)	Ru1—N1A—C1A—C2A	176.9 (5)
N2—Ru1—N3—C13	54.4 (5)	C3A—C2A—C1A—N1A	1.9 (12)
N1A—Ru1—N3—C13	−129.4 (5)	C1A—C2A—C3A—C4A	−2.5 (12)
N3A—Ru1—N3—C13	−43.4 (5)	C12A—C4A—C3A—C2A	0.4 (11)
N1—Ru1—N3—C17	−42.1 (5)	C5A—C4A—C3A—C2A	−175.7 (8)
N2—Ru1—N3—C17	−121.8 (5)	C10A—C9A—C8A—C7A	0.8 (14)
N1A—Ru1—N3—C17	54.4 (5)	C6A—C7A—C8A—C9A	−179.7 (8)
N3A—Ru1—N3—C17	140.4 (4)	C11A—C7A—C8A—C9A	−0.4 (11)
C17A—N3A—C13A—C14A	0.9 (10)	N4A—C15A—C16A—C17A	176.6 (7)
Ru1—N3A—C13A—C14A	171.7 (5)	C14A—C15A—C16A—C17A	−3.8 (12)
N2A—C11A—C7A—C8A	0.9 (9)	N3A—C17A—C16A—C15A	3.6 (11)
C12A—C11A—C7A—C8A	−178.4 (6)	N1—C12—C4—C3	−1.3 (11)
N2A—C11A—C7A—C6A	−179.7 (6)	C11—C12—C4—C3	177.3 (7)
C12A—C11A—C7A—C6A	1.0 (9)	N1—C12—C4—C5	179.2 (7)
C1A—N1A—C12A—C11A	175.6 (6)	C11—C12—C4—C5	−2.1 (10)
Ru1—N1A—C12A—C11A	−1.0 (6)	C12—C4—C3—C2	−0.3 (13)
C1A—N1A—C12A—C4A	−3.3 (8)	C5—C4—C3—C2	179.1 (9)
Ru1—N1A—C12A—C4A	−179.9 (5)	C11A—N2A—C10A—C9A	2.1 (11)
N2A—C11A—C12A—N1A	1.2 (8)	Ru1—N2A—C10A—C9A	−179.9 (7)
C7A—C11A—C12A—N1A	−179.5 (6)	C8A—C9A—C10A—N2A	−1.7 (14)
N2A—C11A—C12A—C4A	−179.9 (5)	C7—C6—C5—C4	0.2 (14)
C7A—C11A—C12A—C4A	−0.5 (9)	C12—C4—C5—C6	1.3 (13)
C8A—C7A—C6A—C5A	178.1 (8)	C3—C4—C5—C6	−178.1 (9)
C11A—C7A—C6A—C5A	−1.2 (11)	C11—N2—C10—C9	0.0 (9)
C13A—N3A—C17A—C16A	−2.0 (10)	Ru1—N2—C10—C9	−179.6 (5)
Ru1—N3A—C17A—C16A	−173.1 (5)	C8—C9—C10—N2	1.3 (11)
N1A—C12A—C4A—C3A	2.7 (9)	C4—C3—C2—C1	2.9 (17)
C11A—C12A—C4A—C3A	−176.2 (6)	N1—C1—C2—C3	−4.2 (16)
N1A—C12A—C4A—C5A	179.0 (6)	C17—N3—C13—C14	−2.1 (8)
C11A—C12A—C4A—C5A	0.2 (9)	Ru1—N3—C13—C14	−178.5 (5)
C1—N1—C12—C4	0.2 (10)	C13—N3—C17—C16	0.7 (9)
Ru1—N1—C12—C4	−179.5 (5)	Ru1—N3—C17—C16	177.3 (5)
C1—N1—C12—C11	−178.5 (6)	N3—C17—C16—C15	0.6 (10)
Ru1—N1—C12—C11	1.8 (7)	N4—C15—C16—C17	176.8 (7)
N2—C11—C12—N1	−0.5 (8)	C14—C15—C16—C17	−0.7 (9)
C7—C11—C12—N1	−179.6 (6)	N3—C13—C14—C15	2.1 (10)
N2—C11—C12—C4	−179.2 (6)	N4—C15—C14—C13	−178.0 (6)
C7—C11—C12—C4	1.6 (9)	C16—C15—C14—C13	−0.6 (9)
C7A—C6A—C5A—C4A	0.8 (12)	C10—C9—C8—C7	−1.0 (11)
C3A—C4A—C5A—C6A	175.6 (7)	C9—C8—C7—C11	−0.5 (11)
C12A—C4A—C5A—C6A	−0.3 (10)	C9—C8—C7—C6	−179.3 (7)
C12—N1—C1—C2	2.5 (12)	N2—C11—C7—C8	1.9 (10)
Ru1—N1—C1—C2	−177.9 (7)	C12—C11—C7—C8	−179.0 (6)
N3A—C13A—C14A—C15A	−1.4 (11)	N2—C11—C7—C6	−179.3 (6)
C7A—C11A—N2A—C10A	−1.7 (9)	C12—C11—C7—C6	−0.2 (9)
C12A—C11A—N2A—C10A	177.6 (6)	C5—C6—C7—C8	178.1 (8)
C7A—C11A—N2A—Ru1	179.9 (5)	C5—C6—C7—C11	−0.7 (11)

### Hydrogen-bond geometry (Å, º)

**Table:**

*D*—H···*A*	*D*—H	H···*A*	*D*···*A*	*D*—H···*A*
N4—H4*A*···F5	0.86	2.45	3.231 (12)	151
N4*A*—H4*A*1···F1*A*^i^	0.86	2.23	3.063 (12)	163
N4*A*—H4*A*2···F3*A*^ii^	0.86	2.34	3.18 (2)	165

Symmetry codes: (i) *x*, −*y*+3/2, *z*−1/2; (ii) *x*+1/2, *y*+1/2, −*z*+3/2.
